# Supplementary material for: Transcriptomic Insights into Mechanisms of Early Seed Maturation in the Garden Pea (Pisum sativum L.)
Source: Cells. 2020 Mar 23;9(3):779. doi: 10.3390/cells9030779 (PMC7140803; doi:10.3390/cells9030779)

Zhewan−1(10) vs Zhon

Global Summary

%DE = 0.9

genes with fdr < 0.2 = 569 (211 + / 358 −)

genes with fdr < 0.1 = 509 (179 + / 330 −)

genes with fdr < 0.05 = 464 (157 + / 307 −)

genes with fdr < 0.01 = 410 (130 + / 280 −)

genes in genesets = 0

<FC> = 0

<t−score> = −13776.4

<p−value> = 0

<fdr> = 0.1

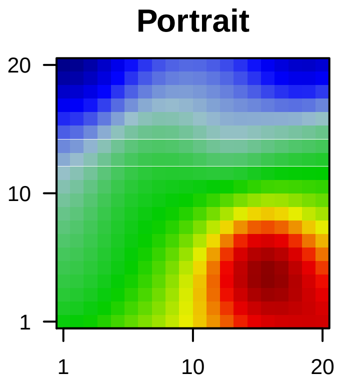

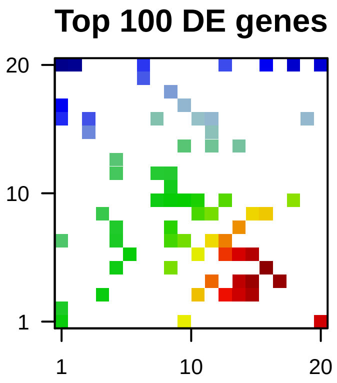

Global Genelist

Rank	ID	log(FC)	p−value	fdr	Metagene	Description
1	TRINITY_DN2	−1.69	0e+00	0.0000000[illegible]	[illegible] x 1	
2	TRINITY_DN6	0.29	0e+00	0.0000000[illegible]	[illegible] x 1	
3	TRINITY_DN7	−1.28	0e+00	0.0000000[illegible]	[illegible] x 20	
4	TRINITY_DN6	0.69	0e+00	0.0000000[illegible]	[illegible] x 20	
5	TRINITY_DN5	−0.2	5e−10	0.0000007[illegible]	[illegible] x 19	
6	TRINITY_DN1	0.22	2e−09	0.0000001[illegible]	[illegible] x 6	
7	TRINITY_DN7	0.14	4e−09	0.0000001[illegible]	[illegible] x 9	
8	TRINITY_DN1	0.07	7e−09	0.0000005[illegible]	[illegible] x 5	
9	TRINITY_DN3	−0.17	9e−09	0.0000003[illegible]	[illegible] x 15	
10	TRINITY_DN4	−2.17	9e−09	0.0000001[illegible]	[illegible] x 20	
11	TRINITY_DN5	−1.04	1e−08	0.0000002[illegible]	[illegible] x 20	
12	TRINITY_DN1	−0.28	2e−08	0.0000002[illegible]	[illegible] x 20	
13	TRINITY_DN5	−0.6	3e−08	0.0000002[illegible]	[illegible] x 20	
14	TRINITY_DN5	0.22	3e−08	0.0000002[illegible]	[illegible] x 4	
15	TRINITY_DN3	0.16	3e−08	0.000002[illegible]	[illegible] x 1	
16	comp11635_c	0	7e−08	0.000002[illegible]	[illegible] x 11	
17	TRINITY_DN2	0.01	1e−07	0.0000040[illegible]	[illegible] x 6	
18	TRINITY_DN3	0.27	2e−07	0.0000040[illegible]	[illegible] x 9	
19	TRINITY_DN1	−0.01	3e−07	0.000004[illegible]	[illegible] x 12	
20	TRINITY_DN6	0.21	3e−07	0.000004[illegible]	[illegible] x 9	

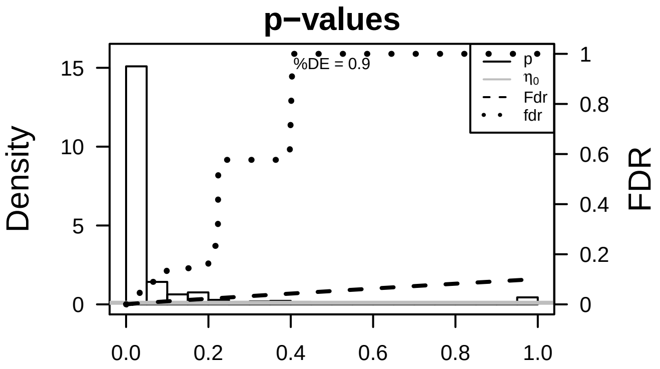

Supplement: Supplementary file 1 [file cells-09-00779-s001.zip › Supplementary materials/FileS3.zip!/FileS2/Hormones - Results/Summary Sheets - Differences/Reports/Zhewan.1.10..vs.Zhongwan.6.25..pdf]
